# Single nucleotide polymorphisms in genes encoding penicillin-binding proteins in β-lactamase-negative ampicillin-resistant *Haemophilus influenzae* in Japan

**DOI:** 10.1186/s13104-018-3169-0

**Published:** 2018-01-20

**Authors:** Kazuhisa Misawa, Norihito Tarumoto, Shinsuke Tamura, Morichika Osa, Takaaki Hamamoto, Atsushi Yuki, Yuji Kouzaki, Kazuo Imai, Runtuwene Lucky Ronald, Toshiyuki Yamaguchi, Takashi Murakami, Shigefumi Maesaki, Yutaka Suzuki, Akihiko Kawana, Takuya Maeda

**Affiliations:** 1 Department of Infectious Diseases and Pulmonary Medicine, National Defense Medical College, Saitama, Japan; 2 Department of Infectious Disease and Infection Control, Saitama Medical University, Saitama, Japan; 3 Center for Clinical Infectious Diseases and Research, Saitama Medical University, Saitama, Japan; 4 Department of Pediatrics, National Defense Medical College, Saitama, Japan; 5 Department of Laboratory Medicine, National Defense Medical College Hospital, Saitama, Japan; 6 Department of Computational Biology and Medical Science, Graduate School of Frontier Sciences, The University of Tokyo, Chiba, Japan; 7 Department of Microbiology, Saitama Medical University, 38 Morohongo, Moroyama-machi, Iruma-gun, Saitama, 350-0495 Japan

**Keywords:** *Haemophilus influenzae*, β-Lactamase-negative ampicillin-resistant (BLNAR), Penicillin binding protein, SNP

## Abstract

**Objective:**

β-Lactamase-negative ampicillin-resistant *Haemophilus influenzae* is a common opportunistic pathogen of hospital- and community-acquired infections, harboring multiple single nucleotide polymorphisms in the *ftsI* gene, which codes for penicillin-binding protein-3. The objectives of this study were to perform comprehensive genetic analyses of whole regions of the penicillin-binding proteins in *H. influenzae* and to identify additional single nucleotide polymorphisms related to antibiotic resistance, especially to ampicillin and other cephalosporins.

**Results:**

In this genome analysis of the *ftsI* gene in 27 strains of *H. influenzae*, 10 of 23 (43.5%) specimens of group III genotype β-lactamase-negative ampicillin-resistant *H. influenzae* were paradoxically classified as ampicillin-sensitive phenotypes. Unfortunately, we could not identify any novel mutations that were significantly associated with ampicillin minimum inhibitory concentrations in other regions of the penicillin-binding proteins, and we reconfirmed that susceptibility to β-lactam antibiotics was mainly defined by previously reported SNPs in the *ftsI* gene. We should also consider detailed changes in expression that lead to antibiotic resistance in the future because the acquisition of resistance to antimicrobials can be predicted by the expression levels of a small number of genes.

**Electronic supplementary material:**

The online version of this article (10.1186/s13104-018-3169-0) contains supplementary material, which is available to authorized users.

## Introduction

The main molecular mechanism of non-β-lactamase resistance among β-lactamase-negative ampicillin-resistant (BLNAR) strains is assumed to be a decreased affinity of penicillin-binding protein (PBP)3 to β-lactams [1–3]. Ubukata et al. proved that amino acid substitutions in the SSN motifs (M377I, S385T, and L389F) and KTG motifs (R517H and N526K) in BLNAR strains are major causes of resistance to ampicillin (ABPC), and that these SNPs lead to changes in the three-dimensional structure of the active binding site of PBP3 [4].

To date, along with the widespread global incidence of BLNAR *H. influenzae*, several isolates have been found to harbor the typical mutation patterns of BLNAR mutants (genotype BLNAR, gBLNAR) with susceptibility to low ABPC minimum inhibitory concentrations (MICs) [5, 6]. Osaki et al. reported elevated cephalosporin MICs in a strain of *H. influenzae* possessing artificially mutated *ftsI* genes, which was expected; however, the ABPC MICs were not elevated, contrary to expectations [7]. These data also suggest that the *ftsI* gene could play a different role in penicillin resistance from its role in resistance to other cephalosporin antibiotics, and that resistance to ABPC is not only determined by the presence or absence of mutations on the *ftsI* gene.

Few studies have investigated the recent trends of genetic polymorphisms in the *ftsI* gene, despite the worldwide spread of BLNAR *H. influenzae*. We hypothesized that novel SNPs on the *ftsI* gene that affect the susceptibility to β-lactams have emerged and could become a threat to the world in the future. The objectives of this study were to perform comprehensive genetic analyses of whole regions of the PBPs in *H. influenzae* and to identify new SNPs related to antibiotic resistance, especially to ABPC and other cephalosporins.

## Main text

### Materials and methods

We collected 39 clinical isolates of *H. influenzae* from January 2014 to March 2016 at the National Defense Medical College Hospital. These isolates were identified using standard microbiological methods to identify *H. influenzae* (rabbit blood agar and conventional X and V factor requirements test) (Eiken Chemical Co., Tokyo, Japan, Cat. No. E-DC08) [8]. Isolates were stored at − 30 °C until use and recovered after inoculation onto chocolate agar (Kyokuto Pharmaceuticals, Tokyo, Japan, Cat. No. 251169) at 37 °C in 5% CO_2_ for 24 h. Genomic DNA was extracted with the EZ Extract for DNA kit (Advanced Microorganism Research, Gifu, Japan, Cat. No. 76815M). MICs of the antibiotics were determined by the broth dilution method (Kyokuto Pharmaceuticals, Cat. Nos. 551-0000-0 and 08930), in accordance with the guidelines of the Clinical Laboratory Standard Institute (document M100-S25) [9]. β-Lactamase production was identified using the nitrocefin method with BBL Cefinase Paper Disc kit (Becton–Dickinson, Franklin Lakes, NJ, Cat. No. 231650).

Extracted DNA specimens subsequently underwent library preparation using TruSeq Nano DNA Sample Preparation Kit (Illumina, San Diego, CA, Cat. Nos. FC-121-4001 and 4002), according to the low sample protocol provided. Single sequencing was performed with an Illumina HiSeq 2500 (Illumina) using 100 bp paired-end runs (Illumina, Cat. Nos. PE-401-3001, FC-401-3001 and FC-121-1003). BWA (v 0.7.15) was used to map raw FASTQ data [10]. The reference sequences used in this study are listed in Additional file 1. Sequences of 16S ribosomal RNA (rRNA), *recA*, *fucK*, *hpd*, and *sodC* were used to distinguish *H. influenzae* from *H. haemolyticus* according to previous reports [11–14]. Capsular formation and serotyping were performed according to the *BexDCBA* and region II in the *cap* locus [15]. Consensus FASTQ data were extracted from the mapping data, and bases with a quality score of less than 20 were eliminated. SNP detection, prediction of amino acid substitutions, and phylogenetic analyses of 16S rRNA and *recA* were performed with MEGA (v 7.0.21) [16]. The presence of *fucK*, *hpd*, and *sodC* was determined from mapping data using Integrative Genomic Viewer (v 2.3.81) [17, 18]. All synonymous SNPs were removed in this study.

Mann–Whitney U tests were used to ascertain the association between the MICs of the antibiotics and the presence or absence of each SNP. Associations were considered statistically significant if the *p* value for any of the SNPs was less than the Bonferroni-adjusted significance threshold. All statistical analyses were calculated with R (v 3.4.0; R Foundation for Statistical Computing, Vienna, Austria [http://www.R-project.org/]) and package “EnvStats” [19].

Based on the *ftsI* gene sequence information, each specimen was classified into one of the following major genotypes: β-lactamase-negative ampicillin-susceptible (gBLNAS), strains without amino acid substitutions in the KTG motif (N526K or R517H) and SSN motif (M377I, S385T, or L389F) in *ftsI*; group I BLNAR, strains with N526K in the KTG motif and no mutations in the SSN motif; group II BLNAR, strains with R517H in the KTG motif and no mutations in the SSN motif; and group III BLNAR, strains with mutations in both the SSN and the KTG motifs [4, 20]. In addition, we defined a strain with the *bla* gene and without amino acid substitutions in the *ftsI* gene as a β-lactamase-positive ampicillin-resistant (BLPAR) strain.

### Results

#### Origins of the clinical samples and β-lactamase producibility

Among the 39 isolates, 18 were extracted from sputum and the rest from nasal discharge (15/39), bronchoalveolar lavage fluid (3/39), pharyngeal swabs (2/39), blood (2/39), and biliary drain fluid (1/39). There were 5 (12.8%) BLPAR strains, and the other 34 were non-producing strains.

#### Antibiotics MICs and genotypes

**Fig. 1:**
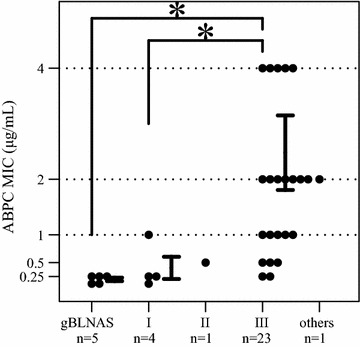
Relationship between non-β-lactamase-producing genotype strains and ABPC MICs. * represents p value < 0.05. I, II, and III represent gBLNAR groups I, II, and III respectively

The MIC profiles of the antibiotics examined in this study are shown in Additional file 2. ABPC MICs were significantly higher in group III BLNAR than in the gBLNAS strains and group I/II gBLNAR strains. However, 10 of 23 specimens in group III BLNAR (43.5%) were paradoxically classified as ABPC-sensitive strains (MIC ≤ 1 µg/mL; Fig. 1).

#### Genetic confirmation of *H. influenzae* and serotyping

Genome analyses of 34 non-β-lactamase-producing strains were performed. All strains were identified as *H. influenzae* and simultaneously distinguished from both *H. parainfluenzae* and *H. haemolyticus* based on the phylogenetic analysis of 16S rRNA and *recA* genes. All 34 strains possessed the *hpd* gene; nevertheless, 2 strains were finally excluded from further SNP analysis because they lacked the *fucK* gene and had the *sodC* gene instead. The reason for their exclusion was that most *H. haemolyticus* strains possess *sodC* gene, which is a characteristic of the species, and *H. influenzae* possesses both *fucK* and *hpd* genes. The remaining 32 strains were confirmed as nontypeable serotypes because they did not have *BexDCBA* and region II in the *cap* locus.

#### SNP analysis of PBP-coding genes

**Fig. 2:**
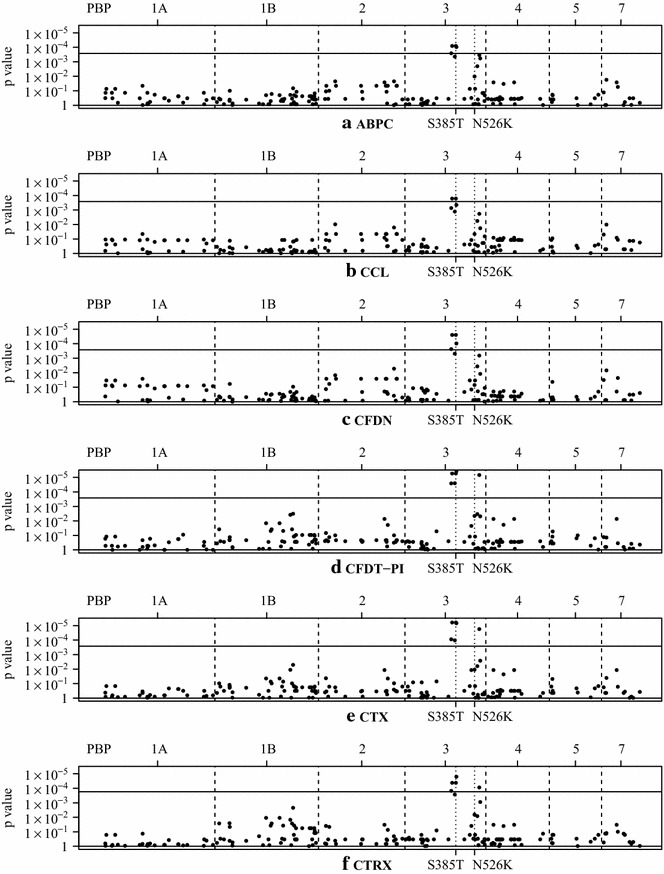
Association between SNPs and MICs. Plot of Mann–Whitney test p values of non-β-lactamase-producing strains. The horizontal axis shows the positions of the SNPs in each PBP coding gene. Dots represent the p values for each SNP. The upper, solid, horizontal lines show the level of significance after adjustment with the Bonferroni method (0.05 divided by 190). The two dotted lines in pbp3 represent the positions of the major SSN and KTG motif mutations: S385T and N526K. **a** ampicillin (ABPC), **b** cefaclor (CCL), **c** cefdinir (CFDN), **d** cefditoren pivoxil (CFDN-PI), **e** cefotaxime (CTX), and **f** ceftriaxone (CTRX)

We targeted 7 distinct genes encoding PBPs (1A, 1B, 2, 3, 4, 5, and 7) according to the NCBI information for *H. influenzae* Rd KW20 (Accession No. NC_000907). Unfortunately, we could not obtain the complete sequences of the PBP-coding genes in 5 of 32 strains, but we successfully completed SNP sequence analysis in 27. As a result of our analysis, we found SNPs with significant associations with the MICs of ABPC on the SSN motif (D350N, S357N, S385T, and L389F) and near the KTG motif (V562L) in the *ftsI* gene (coding PBP-3). Figure 2 illustrates the associations between the p-values derived from Mann–Whitney U tests of non-β-lactamase-producing strains for the *ftsI* gene and the SNPs that show significant associations with the MICs of ABPC. We also found SNPs with low p-values on the SSN motif (M377I) and near the KTG motif (V562L) in the *ftsI* gene (coding PBP-3). No novel SNPs were found in the other PBP-coding genes (Fig. 2a). The MICs of the other cephalosporin antibiotics were similar to those of ABPC (Fig. 2b–f). Additionally, these SNPs showed strong positive correlations with each other (Table 1).

**Table 1:** Correlation coefficients between 2 SNPs in the *ftsI* gene

	D350N	S357N	M377I	S385T	L389F	V562L
D350N		0.924	0.924	0.924	0.795	0.739
S357N			0.846	1.000	0.860	0.798
M377I				0.846	0.860	0.798
S385T					0.860	0.798
L389F						0.928
V562L						

#### Analysis of *acrA*, *acrB*, *acrR*, and *tolC* genes

The sequences of *acrA, acrB, acrR,* and *tolC* genes were also analyzed in 27 samples. Whereas 16 samples had about 46 bp deletions in the *acrB* gene according to the mapping data, no significant changes in the MICs of the antibiotics were identified in either the presence or absence of the *acrB* mutations (Additional file 3).

### Discussion

In this study, our data reconfirmed the significant associations between SNPs located in the *ftsI* gene and the MICs of ABPC or other cephalosporins in β-lactamase-negative *H. influenza*e; however, no other novel SNPs could be identified. We also found that group III BLNAR strains of *H. influenzae* with low ABPC MICs could paradoxically harbor typical SNPs in the *ftsI* gene. It was also suggested that discrepancies between the genotypes and phenotypes among BLNAR strains have already become commonplace in Japan. We identified that about up to 40% of isolates within group III gBLNAR genotype had low ABPC MICs, and these were classed as ABPC-sensitive phenotype.

We considered several reasons for the discrepancies between phenotypes and genotypes. First, MICs from the disk-testing method are considered to be influenced by the amount of bacteria in *H. influenzae* and our data reaffirmed the difficulty of predicting ABPC MICs based on the mutation pattern of the *ftsI* gene [21]. Second, ABPC affinity for PBPs was different from that of the other cephalosporins. Cefotaxime and other cephalosporins have the highest affinity for PBP2, whereas ampicillin had higher affinity for PBP1A and PBP4 [4]. Our data showed that cephalosporin agents tended to have a higher association with the MICs than ABPC. These findings confirm that the active sites of PBPs depend on the antibiotic agents. Third, subtle differences among clusters of species might be one of the reasons for the discrepancies between phenotypes and genotypes in clinical practice. Among the 27 isolates of *H. influenzae* identified via conventional biochemical methods, 2 were lacking the *fucK* gene and possessed the *sodC* gene instead, which is a typical profile of *H. haemolyticus* and not *H. influenzae*. Conventionally, the identification of *H. influenzae* is determined based on the results of hemolysis in rabbit blood agar and the conventional X and V factor requirements test. However, *H. haemolyticus* strains could be misdiagnosed as *H. influenzae* using these conventional protocols in clinical practice. Furthermore, even 16S rRNA phylogenetic analysis is not enough to distinguish these two species, and some of them can be categorized as “fuzzy species” [13, 14, 22–24]. Although β-lactamase resistance in *H. haemolyticus* is similar to that in *H. influenzae*, detailed information about mutations in the *ftsI* gene and the sensitivity of *H. haemolyticus* to ABPC and fuzzy species remains limited [25].

Some reports have also suggested that efflux pumps are involved in the development of antibiotic resistance, including to ABPC [26–28]. Kaczmarek et al. indicated that both the *ftsI* and *acrR* genes are associated with the antibiotic efflux pump and frameshift mutations, especially in *acrR*, could promote high ABPC MICs by accelerating AcrAB-mediated efflux [29]. However, our data did not show significant changes in the MICs either in the presence or absence of the *acrB* mutation [30].

## Conclusion

It is possible that the gBLNAR strains could spread more widely in communities than can be estimated from MIC values. Unfortunately, we could not exclude the possibility that other mechanisms independent of the SNPs could contribute to the elevations in the MICs of the antibiotics. Therefore, continuous studies are crucial in order to gain an understanding of the molecular basis of β-lactam antibiotic resistance in BLNAR *H. influenzae*. Furthermore, we should consider in detail the changes in expression that lead to antibiotic resistance in our future plans because the acquisition of resistance to antimicrobials can also be predicted by the levels of expression of a small number of genes.

## Limitations

First, our findings demonstrated that SNPs located in the SSN motif in the *ftsI* gene correlated highly with each other and we could not conclude which of the three types of SNPs in the SSN motif contributed most to the MICs of the antibiotics. Furthermore, we cannot rule out the possibility that horizontal transfer of a resistance gene could be involved [31]. Second, variations among the mutations were limited due to the small number of samples. Third, β-lactamase-positive amoxicillin/clavulanate resistant strains of *H. influenzae* have recently emerged in clinical settings [32]. However, we did not include these strains because only a few were found in this study.

## Additional files

**Additional file 1.** Reference sequences used in this study.

**Additional file 2.** Antibiotic minimum inhibitory concentrations (MICs) for β-lactamase non-producing *H. influenzae* examined in this study.

**Additional file 3.** *AcrB* mutations and their relationship with ABPC MICs for each genotype.

## Abbreviations

ABPC: ampicillin
BLNAR: β-lactamase-negative ampicillin-resistant
BLNAS: β-lactamase-negative ampicillin-susceptible
BLPAR: β-lactamase-producing ampicillin-resistant
MICs: minimum inhibitory concentrations
PBP: penicillin-binding protein
rRNA: ribosomal RNA
SNP: single nucleotide polymorphisms

## References

[1] Mendelman PM, Chaffin DO, Stull TL, Rubens CE, Mack KD, Smith AL (1984). Characterization of non-β-lactamase-mediated ampicillin resistance in *Haemophilus influenzae*. Antimicrob Agents Chemother.

[2] Tristram S, Jacobs MR, Appelbaum PC (2007). Antimicrobial resistance in *Haemophilus influenzae*. Clin Microbiol Rev.

[3] Skaare D, Anthonisen IL, Caugant DA, Jenkins A, Steinbakk M, Strand L (2014). Multilocus sequence typing and *ftsI* sequencing: a powerful tool for surveillance of penicillin-binding protein 3-mediated beta-lactam resistance in nontypeable *Haemophilus influenzae*. BMC Microbiol.

[4] Ubukata K, Shibasaki Y, Yamamoto K, Chiba N, Hasegawa K, Takeuchi Y (2001). Association of amino acid substitutions in penicillin-binding protein 3 with β-lactam resistance in β-lactamase-negative ampicillin-resistant *Haemophilus influenzae*. Antimicrob Agents Chemother.

[5] Lâm TT, Claus H, Elias J, Frosch M, Vogel U (2015). Ampicillin resistance of invasive *Haemophilus influenzae* isolates in Germany 2009–2012. Int J Med Microbiol.

[6] Dabernat H, Delmas C, Seguy M, Pelissier R, Faucon G, Bennamani S (2002). Diversity of β-lactam resistance-conferring amino acid substitutions in penicillin-binding protein 3 of *Haemophilus influenzae*. Antimicrob Agents Chemother.

[7] Osaki Y, Sanbongi Y, Ishikawa M, Kataoka H, Suzuki T, Maeda K (2005). Genetic approach to study the relationship between penicillin-binding protein 3 mutations and *Haemophilus influenzae* β-lactam resistance by using site-directed mutagenesis and gene recombinants. Antimicrob Agents Chemother.

[8] Garcia LS (2010). Clinical microbiology procedures handbook.

[9] Clinical and Laboratory Standards Institute (2015). Performance standards for antimicrobial susceptibility testing: twenty-fifth informational supplement M100-S25.

[10] Li H, Durbin R (2009). Fast and accurate short read alignment with Burrows-Wheeler Transform. Bioinformatics.

[11] Theodor MJ, Anderson RD, Wang X, Katz LS, Vuong JT, Bell ME (2012). Evaluation of new biomarker genes for differentiating *Haemophilus influenzae* from *Haemophilus haemolyticus*. J Clin Microbiol.

[12] Nørskov-Lauritsen N, Overballe MD, Kilian M (2009). Delineation of the species *Haemophilus influenzae* by phenotype, multilocus sequence phylogeny, and detection of marker genes. J Bacteriol.

[13] McCrea KW, Xie J, LaCross N, Patel M, Mukundan D, Murphy TF (2008). Relationships of nontypeable *Haemophilus influenzae* strains to hemolytic and nonhemolytic *Haemophilus haemolyticus* strains. J Clin Microbiol.

[14] Binks MJ, Temple B, Kirkham LA, Wiertsema SP, Dunne EM, Richmond PC (2012). Molecular surveillance of true nontypeable *Haemophilus influenzae*: an evaluation of PCR screening assays. PLoS ONE.

[15] Maaroufi Y, Bruyne JMD, Heymans C, Crokaert F (2007). Real-time PCR for determining capsular serotypes of *Haemophilus influenzae*. J Clin Microbiol.

[16] Kumar S, Stecher G, Tamura K (2016). MEGA7: Molecular Evolutionary Genetics Analysis Version 7.0 for bigger datasets. Mol Biol Evol..

[17] Robinson James T, Thorvaldsdóttir Helga, Winckler Wendy, Guttman Mitchell, Lander Eric S, Getz Gad, Mesirov Jill P (2011). Integrative Genomics Viewer. Nat Biotechnol.

[18] Thorvaldsdóttir Helga, Robinson James T, Mesirov Jill P (2013). Integrative Genomics Viewer (IGV): high-performance genomics data visualization and exploration. Brief Bioinform.

[19] Millard SP (2013). EnvStats: an R package for environmental statistics.

[20] Hotomi M, Fujihara K, Billal DS, Suzuki K, Nishimura T, Baba S (2007). Genetic characteristics and clonal dissemination of β-lactamase-negative ampicillin-resistant *Haemophilus influenzae* strains isolated from the upper respiratory tract of patients in Japan. Antimicrob Agents Chemother.

[21] Ubukata K, Chiba N, Hasegawa K, Shibasaki Y, Sunakawa K, Nonoyama M (2002). Differentiation of beta-lactamase-negative ampicillin-resistant *Haemophilus influenzae* from other *H. influenzae* strains by a disc method. J Infect Chemother.

[22] Anderson R, Wang X, Briere EC, Katz LS, Cohn AC, Clark TA (2012). *Haemophilus haemolyticus* isolates causing clinical disease. J Clin Microbiol.

[23] Price EP, Sarovich DS, Nosworthy E, Beissbarth J, Marsh RL, Pickering J (2015). *Haemophilus influenza*e: using comparative genomics to accurately identify a highly recombinogenic human pathogen. BMC Genom.

[24] de Gier C, Kirkham LA, Nørskov-Lauritsen N (2015). Complete deletion of the fucose operon in *Haemophilus influenzae* is associated with a cluster in multilocus sequence analysis-based phylogenetic group II related to *Haemophilus haemolyticus*: implications for identification and typing. J Clin Microbiol.

[25] Witherden EA, Tristram SG (2013). Prevalence and mechanisms of β-lactam resistance in *Haemophilus haemolyticus*. J Antimicrob Chemother.

[26] Sánchez L, Pan W, Viñas M, Nikaido H (1997). The *acrAB* homolog of *Haemophilus influenzae* codes for a functional multidrug efflux pump. J Bacteriol.

[27] Dean CR, Narayan S, Daigle DM, Dzink-Fox JL, Puyang X, Bracken KR (2015). Role of the *acrAB*-*tolC* efflux pump in determining susceptibility of *Haemophilus influenzae* to the novel peptide deformylase inhibitor LBM415. Antimicrob Agents Chemother.

[28] Trepod CM, Mott JE (2004). Identification of the *Haemophilus influenzae tolC* gene by susceptibility profiles of insertionally inactivated efflux pump mutants. Antimicrob Agents Chemother.

[29] Kaczmarek FS, Gootz TD, Dib-Haji F, Shang W, Hallowel S, Cronan M (2004). Genetic and molecular characterization of β-lactamase negative ampicillin resistant *Haemophilus influenzae* with unusually high resistance to ampicillin. Antimicrob Agents Chemother.

[30] Seyama S, Wajima T, Nakaminami H, Noguchi N (2016). Molecular mechanism of epidemic clarithromycin-resistant β-lactamase-non-producing ampicillin-resistant *Haemophilus influenzae* in Japan. Antimicrob Agents Chemother.

[31] Witherden EA, Bajance-Lavado MP, Tristram SG, Nunes A (2014). Role of inter-species recombination of the *ftsI* gene in the dissemination of altered penicillin-binding-protein-3-mediated resistance in *Haemophilus influenzae* and *Haemophilus haemolyticus*. J Antimicrob Chemother.

[32] Doren GV, Brueggemann AB, Pierce G, Jolley HP, Rauch A (1997). Antibiotic resistance among clinical isolates of *Haemophilus influenzae* in the United States in 1994 and 1995 and detection of β-lactamase-positive strains resistant to amoxicillin-clavulanate: results of a national multicenter surveillance study. Antimicrob Agents Chemother.

