# Supplementary material for: Single nucleotide polymorphisms in genes encoding penicillin-binding proteins in β-lactamase-negative ampicillin-resistant Haemophilus influenzae in Japan
Source: BMC Res Notes. 2018 Jan 20;11:53. doi: 10.1186/s13104-018-3169-0 (PMC5775570; doi:10.1186/s13104-018-3169-0)
Supplement: Supplementary file 1 — Additional file 1. Reference sequences used in this study. [file 13104_2018_3169_MOESM1_ESM.docx]

**Additional Material 1.** Reference sequences used in this study.

| **Species (strain)** | **Gene**  **or protein** | **Accession no.** | **Nucleotide position** | **Reference** |
| --- | --- | --- | --- | --- |
| *Haemophilus influenzae* (Rd KW20) | pbp1A | NC_000907 | 460645-463239 | NCBI database* |
|  | pbp1B |  | 1796174-1798519 |  |
|  | pbp2 |  | 33446-35401 |  |
|  | pbp3 (*ftsI*) |  | 1197840-1199672 |  |
|  | pbp4 |  | 1407458-1408897 |  |
|  | pbp5 |  | 30229-31410 |  |
|  | pbp7 |  | 387648-388526 |  |
|  | *acrR* |  | 946725-947288 |  |
|  | *acrA* |  | 947368-948516 |  |
|  | *acrB* |  | 948516-951614 |  |
|  | *tolC* |  | 1544120-1545439 |  |
|  | *fucK* |  | 643894-645306 |  |
|  | *hpd* |  | 732946-734040 |  |
|  | 16S rRNA |  | 127172-128720 |  |
|  | *recA* |  | 621492-622556 |  |
| *H. influenzae* | *sodC* | M84012 | 1-799 | NCBI database* |
| *H. influenzae* | *bexDCBA* | X54987 | 1-4005 | [19] |
| *H. influenzae* | serotype a *cap* locus region II | Z37516 | 1-5882 | [19] |
| *H. influenzae* | serotype b *cap* locus | X78559 | 1-8640 | [19] |
| *H. influenzae* (RM127) | serotype c capsular DNA | Z33387 | 1-122 | [19] |
| *H. influenzae* (RM128) | serotype d capsular DNA | Z33389 | 1-155 | [19] |
| *H. influenzae* (RM129) | serotype e capsular DNA | Z33390 | 1-75 | [19] |
| *H. influenzae* (70022) | serotype f capsular locus | AF549211 | 7744-16028 | [19] |

* National Center for Biotechnology Information database: <https://www.ncbi.nlm.nih.gov/>
