# Supplementary material for: Single nucleotide polymorphisms in genes encoding penicillin-binding proteins in β-lactamase-negative ampicillin-resistant Haemophilus influenzae in Japan
Source: BMC Res Notes. 2018 Jan 20;11:53. doi: 10.1186/s13104-018-3169-0 (PMC5775570; doi:10.1186/s13104-018-3169-0)
Supplement: Supplementary file 2 — Additional file 2. Antibiotic minimum inhibitory concentrations (MICs) for β-lactamase non-producing H. influenzae examined in this study. [file 13104_2018_3169_MOESM2_ESM.docx]

**Additional Material 2.** Antibiotic minimum inhibitory concentrations (MICs) for β-lactamase non-producing *H. influenzae* examined in this study*.*

| Antibiotics | MIC_50_ (μg/mL) | MIC_90_ (μg/mL) | MIC range (μg/mL) |
| --- | --- | --- | --- |
| Ampicillin | 1 | 4 | 0.12-16 |
| Cefaclor | 4 | 32 | 0.5-64 |
| Cefdinir | 1 | 8 | 0.03-8 |
| Cefditoren-Pivoxil | 0.12 | 0.5 | 0.03-4 |
| Ceftriaxone | 0.375 | 1 | 0.06-1 |
| Cefotaxime | 0.12 | 0.25 | 0.06-0.5 |
