# Supplementary material for: Single nucleotide polymorphisms in genes encoding penicillin-binding proteins in β-lactamase-negative ampicillin-resistant Haemophilus influenzae in Japan
Source: BMC Res Notes. 2018 Jan 20;11:53. doi: 10.1186/s13104-018-3169-0 (PMC5775570; doi:10.1186/s13104-018-3169-0)
Supplement: Supplementary file 3 — Additional file 3. AcrB mutations and their relationship with ABPC MICs for each genotype. [file 13104_2018_3169_MOESM3_ESM.docx]

**Additional Material 3.** *AcrB* mutations and the relationship with ABPC MIC for each genotype.

| **Genotype** | ***acrB* Mutation** | | |
| --- | --- | --- | --- |
|  | **Present** | **Absent** | **p-value*** |
| gBLNAS | 3 | 2 | 0.739 |
| Group I/II gBLNAR | 3 | 3 | 0.657 |
| Group III gBLNAR | 10 | 13 | 0.828 |

* Mann–Whitney U test was performed to compare the MICs in each genotype group.
